# CardioNet: A human metabolic network suited for the study of cardiomyocyte metabolism

**DOI:** 10.1186/1752-0509-6-114

**Published:** 2012-08-29

**Authors:** Anja Karlstädt, Daniela Fliegner, Georgios Kararigas, Hugo Sanchez Ruderisch, Vera Regitz-Zagrosek, Hermann-Georg Holzhütter

**Affiliations:** 1 Institute of Biochemistry, Charité-Universitätsmedizin Berlin, 10117 Berlin, Charitéplatz 1/ Virchowweg 6, Germany; 2 Center for Cardiovascular Research, Charité-Universitätsmedizin Berlin, 10115 Berlin, Hessische Straße 3-4, Germany

**Keywords:** Computational biology, Flux balance, Heart, Cardiomyocyte, Efficency, Metabolism

## Abstract

**Background:**

Availability of oxygen and nutrients in the coronary circulation is a crucial determinant of cardiac performance. Nutrient composition of coronary blood may significantly vary in specific physiological and pathological conditions, for example, administration of special diets, long-term starvation, physical exercise or diabetes. Quantitative analysis of cardiac metabolism from a systems biology perspective may help to a better understanding of the relationship between nutrient supply and efficiency of metabolic processes required for an adequate cardiac output.

**Results:**

Here we present CardioNet, the first large-scale reconstruction of the metabolic network of the human cardiomyocyte comprising 1793 metabolic reactions, including 560 transport processes in six compartments. We use flux-balance analysis to demonstrate the capability of the network to accomplish a set of 368 metabolic functions required for maintaining the structural and functional integrity of the cell. Taking the maintenance of ATP, biosynthesis of ceramide, cardiolipin and further important phospholipids as examples, we analyse how a changed supply of glucose, lactate, fatty acids and ketone bodies may influence the efficiency of these essential processes.

**Conclusions:**

CardioNet is a functionally validated metabolic network of the human cardiomyocyte that enables theorectical studies of cellular metabolic processes crucial for the accomplishment of an adequate cardiac output.

## Background

Cardiovascular diseases are the main cause of death worldwide [1]. The myocardium, comprised of cardiomyocytes, has to fulfil a wide range of metabolic functions serving cellular integrity and energy demand to maintain contractile activity for the cardiac cycle. Consequently, alterations in the metabolism of cardiomyocytes have a great impact on the cyclical contraction of the heart.

More insights into the metabolic changes and efficiency of cardiomyocytes under conditions of heart failure and myocardial hypertrophy may improve treatments of these diseases. A prerequisite for such an approach is the reconstruction of the metabolic network of the human cardiomyocyte. Previous genome-scale metabolic network reconstructions [2-4] have shown their capacity to enable an insight into metabolic changes in altered extra- and intracellular conditions. Computational methods [5-8] offer the possibility to simulate metabolic responses in restricted substrate supply or inhibition of enzymatic reactions observed in diabetes, obesity, starvation and cardiovascular diseases. Most importantly the metabolic efficiency of cardiomyocytes to maintain mechanism, which are directly or indirectly involved in cardiac contraction could be studied, including the synthesis of filament proteins, ion channels and membrane phospholipids as well as energy production and storage. The metabolic efficiency of cardiomyocytes to maintain continuous ATP demand for muscular contraction, replenish and eventually increase, the biosynthesis of macromolecules depends upon the availability of oxygen and external substrates, including fatty acids, glucose, lactate, pyruvate and amino acids [9]. Among these, fatty acids are the preferred substrate, however the contribution of each substrate to metabolic processes is greatly dependent on the cellular state (cell cycle), oxygen supply and endocrinological conditions [10,11]. Recent investigations demonstrated that the fatty acid composition of myocardial membrane phospholipids alters with dietary uptake of fatty acids and oxygen supply [12,13]. An increased incorporation of long-chain omega-3 polyunsaturated fatty acids (e.g. eicosapentaenoic acid, docosahexaenoic acid), mostly derived from dietary essential alpha-linoleic acid (18:3 n-3, ALA) [14], seems to have beneficial effects against the risk of primary cardiac arrest [15,16]. Furthermore structural adaptation to hypoxia [12], thus offers protection from oxidative damage.

Diabetes and obesity have been especially shown to be associated with oxidative stress [17] and damage by reactive oxygen species (ROS) to proteins, nucleic acids and phospholipids such as cardiolipin [18]. The mitochondrial membrane of cardiomyocytes comprises an extraordinary high content of cardiolipin, which cannot be replaced by other phospholipids [19]. Furthermore, cardiolipin is indispensable for the activity of rate-limiting protein complexes in mitochondrial ATP production such as adenine nucleotide translocase [20], F0F1-ATPase [21] or complex I. The latter is considered a source of cellular ROS, which in turn can induce cardiolipin damage and, consequently, decrease cardiac functionality [18].

In addition to permanent utilization of substrates, cardiomyocytes are able to store energy-rich substrates, which prevents myocardial injury in hypoxic or ischemic conditions. An important short-term store is glycogen. The formation is strongly dependent upon nutritional composition of the blood. In particular, a reduction of free fatty acids in plasma by nicotinic acid treatment was found to lower levels of cardiac glycogen in rats [22]. This remarkable finding, considering that glycogen cannot be formed from free fatty acids, underlines the importance of a holistic approach to the study of cardiomyocyte metabolism taking into account all metabolic processes involved in glycogen synthesis.

The aim of the present investigation was to reconstruct a genome-scale metabolic network of the human cardiomyocyte. Using methods of constraint-based optimization, we demonstrate how changes in oxygen and substrate supply influence the efficiency of selected metabolic functions of cardiomyocytes and provide valuable suggestions for substrate compositions allowing optimal accomplishment of metabolic functions to provide cellular integrity and maintain cardiac work.

## Results

First, we reconstructed the metabolic network of the human cardiomyocyte (CardioNet) and tested physiological functions of the cardiomyocyte to ensure full functionality and consistency of the network. Furthermore, we compared our network to two previously reported mitochondrial networks [23,24] and one genome-scale reconstruction of the human heart [25].

Second, we determined substrate and oxygen requirement in varied availability of four different substrates while satisfying a baseline ATP consumption rate. We further analysed the efficiency of these varied substrate compositions and compared our findings to experimental results.

Finally, we performed an extensive simulation of varied availability for nine different substrates while satisfying a complex target function of the cardiomyocyte and analysed the efficiency.

### Metabolic network for the human cardiomyocyte

We developed a fully compartmentalized network of the human cardiomyocyte, which accomplishes various sets of physiological functions of the human heart. The network totals 1793 reactions, including 560 transport reactions and 728 metabolites assigned to 6 different compartments: extracellular, cytosol, mitochondrion, microsome, lysosome and peroxisome (see Table 1). At the current state the synthesis of biopolymers such as DNA and RNA is not part of the metabolic network, therefore no reactions were considered in the network reconstruction which are localisied in the nucleus, e.g. DNA transcription and translation. Instead we restrain the objectives of the metabolic network to the production of building blocks, e.g. amino acids and nucleotides. Any changes in enzyme activity or occurrence, such as in e.g. heart failure, have to be included as constraints into the optimization problem.

**Table 1:** Overview of the metabolic network of human cardiomyocyte

	**Compartments**	**Reactions**	**Transporters**	**Metabolites**	**Literature references**	**Genes**
**amount**	6	1793	560	728	363	2565

Furthermore, the network includes nine generic metabolites (pooled metabolites, [4]) which describe compounds with variable composition and appear only in reactions for lipoprotein particles, such as LDL particles (see Additional file 1).

Moreover, we included the metabolism of distinct fatty acids, glycerolipids, glycerophospholipids and sphingolipids, as far as we could find biochemical evidence for their occurrence in the human heart (see Additional file 2), as well as cross-references from online databases, such as Lipid Maps Classification System [26] and Human Metabolome Database [27].

To ensure consistency and full functionality of the metabolic network, we performed a testing of physiological functions based on knowledge of the cardiac metabolism by using flux balance analysis (FBA) (see Methods). The required functions included phospholipid synthesis (e.g. cardiolipin), conversion of amino acids into citric acid cycle intermediates by transamination or oxidative deamination as well as nucleotide synthesis (see Table 2, Additional file 3 and 4).

**Table 2:** Metabolic and physiological functions tested for the metabolic network

**Classification**		**Metabolic function**	**Cellular function**	**Reference**
**1. Carbohydrates**				
	∙ Monosaccharides	Glucose and fructose metabolism	Energy production	[28,29]
		Glycogen formation	short-term energy storage	[22]
		Ribose	Energy production	[30,31]
			Formation of ribonucleotides	
**2. Carboxylic acids**		Degradation of ketone bodies	Energy production during fasting	[32,33]
			and diabetes	
**3. Lipids**				
	∙ Cholesterol	De novo synthesis (cytosol, peroxisome)	Membrane synthesis	[34,35]
	∙ Fatty acids	Formation of (semi)-essential fatty acids	Membrane synthesis	[36,37]
		*β*-oxidation of (non)-essential fatty acids	Energy production	[38-40]
	∙ Triacylglycerides	De novo synthesis/ degradation of Mono-,	Membrane synthesis	[41,42]
		Di- and Triacylglycerides		
	∙ Phospholipids	De novo synthesis/ degradation of:	Membrane formation	[15,16,41,43-45]
		∘ Phosphatidylserines		
		∘ Phosphatidylcholines		
		∘ Lysophosphatidylcholines		
		∘ Phosphatidylethanolamines		
		∘ Phosphatidylinositol		
		∘ Sphingomyelin		
		∘ Cardiolipin		
	∙ Sphingolipids	Ceramides	Membrane formation, apoptosis	[46]
**4. Proteins**				
	∙ Amino acids	Formation of (non)-essential amino acids	Precursors of cellular proteins, nucleic acids,	[47,48]
			glutathione and thioredoxin	
		Degradation of (non)-essential amino acids	Amino acid homoeostasis,	[49]
			anaplerotic reactions of TCA cycle	
		Glutamine formation	Ammonia detoxification, Protein	[50]
			de novo synthesis	
		De novo synthesis of L-Carnitine	Transport of fatty acids from cytosol into	[51,52]
			mitochondria during *β*-oxidation	
	∙ Tripeptide	De novo synthesis of Glutathione	Prevention of cellular damage due to ROS	[53]
	∙ Polyamines	Formation/ degradation of Prutescine and Spermidine	Cell growth and division	[54,55]
	∙ Proteins	De novo synthesis of:		
		Myosin, Titin, *α*-Sarcoglycan, Tropomyosin, Troponin T	Contractile apparatus, enabling muscular contraction	[56,57]
		De novo synthesis of Thioredoxin	Prevention of cellular damage due to ROS	[47,48]
**5. Nucleic acids**				
	∙ Nucleobases	De novo synthesis/ degradation of purine	Precursors of nucleosides, deoxy-ribonulceotides	[58,59]
		and pyrimidine nucleotides	and ribonucleotides	
		Salvage of purine and pyrimidine nucleotides	Maintaining energy state	[60]
		De novo synthesis/ rephosphorylation of:		
		∘ nucleosides (ATP, CTP, GTP, TTP, UTP)	Energy production for muscular contraction	[59,61-63]
		∘ NADH, NADPH	Energy production and providing redox-state	[64,65]

The import of metabolites during these simulations was restricted to oxygen, glucose, lactate, ketone bodies (acetoacetate, (R)-3-hydroxybutanoate), essential amino and fatty acids as well as vitamins, while the release of intermediates were restricted to metabolic end products, e.g. lactate and glutamine (see Additional 3). In case the network failed to fulfil a required function, we critically evaluated related reactions and metabolites for producibility.

Primary missing intracellular transport reactions and incomplete pathways (e.g. lipid metabolism) were revealed, which required further manual literature review to complete the network functionality. We revised reactions and eventually included new reactions into the metabolic network based on additional evidence for occurrence in the human cardiomyocyte from e.g. experimental studies (see Additional file 2). The resulting metabolic network (CardioNet) and a complete list of all metabolites is provided with the Additional file (see Additional file 1 and 5).

In a next step we compared the metabolic network to previously reported reconstructions of the mitochondrion in cardiomyocytes [23,24] and one genome-scale reconstruction of the human heart [25]. All models used different types of evidence for the network reconstruction process (e.g. transcriptomic, metabolomic, proteomic data) similar to this study. In contrast both mitochondrial networks [23,24] and this study are cross-references from experimental studies or other database not provided in the genome-scale reconstuction of the human heart [25]. We found a large alignment between CardioNet and both mitochondrial networks with 90.48% and 92.49% of the mitochondrial network reactions are represented in CardioNet. In contrast to our model heme biosynthesis is considered to more detail in these networks and arginase II reaction is included. There is evidence for absent expression of arginase II in the normal human cardiomyocyte [66] with up-regulation only during pathological states such as heart failure [67]. The current network reconstruction is based on evidence in the normal human cardiomyocyte, thus reactions (e.g. arginase II) which belong to genes which are not expressed normally, have to be included into the network to study specific pathological conditions. The metabolic network of the human cardiomyocyte (CardioNet) considers 228 additional mitochondrial reactions which are not part of the previous mitochondrial network reconstructions. Our model describes the metabolism of 26 fatty acids and the biosynthesis of important phospholipids such as cardiolipin, phosphatidylserine and phosphatidylcholine. Furthermore, the amino acid metabolism is included to greater extent such as the synthesis of non-essential amino acids (e.g. glycine) which is not part of the mitochondrial networks.

Finally, we compared CardioNet to a genome-scale reconstruction of the human heart [25]. Although the fatty acid metabolism is represented in the human heart model to a greater extent compared to the mitochondrial networks [23,24], important phospholipids such as cardiolipin and the cholesterol biosynthesis in the peroxisome are missing. We found no localisation of citric acid cycle compounds in the mitochondrion such as fumarate and succinate which only occur in the cytosol. Moreover, we applied the presented physiological functions of the cardiomyocyte as part of the network reconstruction process (see Additional file 6) to test the partial network of the human heart [25]. From 110 tested functions 53 had no feasible solution, this included important cellular functions such as the citric acid cycle. Our findings are in concordance with previous studies showing that automatisied network reconstructions based on Recon1 not necessarily lead to a functional network [4,68].

### Calculation of substrate and oxygen uptake rates for ATP consumption in varied substrate availability

We analysed the efficiency of cardiac metabolism in altered substrate supply by applying the metabolic network. The dephosphorylation of ATP through myosin light chain kinase is an important step in the cross-bridge cycle to generate cardiac contraction [56,57]. This dynamic process is not described in the metabolic network. Therefore we demanded a baseline ATP consumption rate (*v*_ATPase_) of 21.6 mmol·min^−1^·(l cell)^−1^[69] to include this important function of the cardiomyocyte.

The target function (*v*_*t*_) for these simulations reads as following:

(1) $$\begin{array}{ll} v_{t}=v_{\text{ATPase}} & \; \end{array}$$

Oxidation of available substrates m should provide sufficient ATP synthesis to enable this ATP consumption rate (*v*_ATPase_). We chose glucose, oleate, acetoacetate and lactate as alternative energy-delivering substrates, that can be taken up and oxidized by cardiomyocytes to generate ATP. The external uptake rate for each substrate is expressed by *v*_*m*_ (*m *= 1,2,*..*,*ns*) and were described as following:

(2) $$\begin{array}{ll} & v_{m}=(\beta_{m}\cdot v_{s})\; \end{array}$$

where the coefficient *β *(0 ≤* β*_*m *_≤ 1) denotes the relative share of the respective substrate m in the total substrate uptake flux *v*_*s*_ (see Methods). We modified the coefficient *β *on a fine grid of values between 0 and 1 and performed in total 176851 flux minimization computations, while minimizing the sum of external uptake fluxes without restricting the oxygen supply (see Additional file 7 for constraints; Additional file 8 and 9 for predicted flux values).

**Figure 1:**
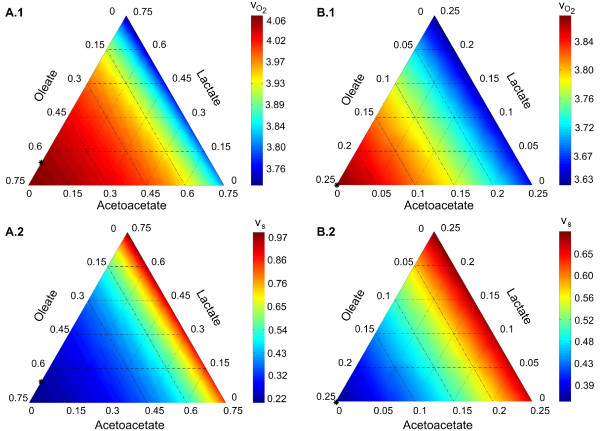
**Illustration of total substrate uptake rate (*v***_***s***_**) and oxygen demand (**$v_{O_{2}}$**) for a fixed glucose supply with (A) *β *= 0.25 and (B) *β *= 0.75.** The ATP consumption rate (*v*_ATPase_) were restricted to 21.6 $\left[\frac{\text{mmol}}{\text{min}\cdot (l\;\text{cell})}\right]$. The panels A.1 and B.1 refer to the oxygen demand ( $\left(v_{O_{2}}\right)$) for the given glucose proportion, while panels A.2 and B.2 refer to the total substrate uptake rate (*v*_*s*_). For each panel we marked the optimal substrate composition (*): **(A)***v*_*Glucose *_= 25%, *v*_*Oleate *_= 64%, *v*_*Acetoacetate *_= 0%, *v*_*Lactate *_= 11%, **(B)***v*_*Glucose *_= 75%, *v*_*Oleate *_= 25%, *v*_*Acetoacetate *_= 0%, *v*_*Lactate *_= 0%.

**Figure 2:**
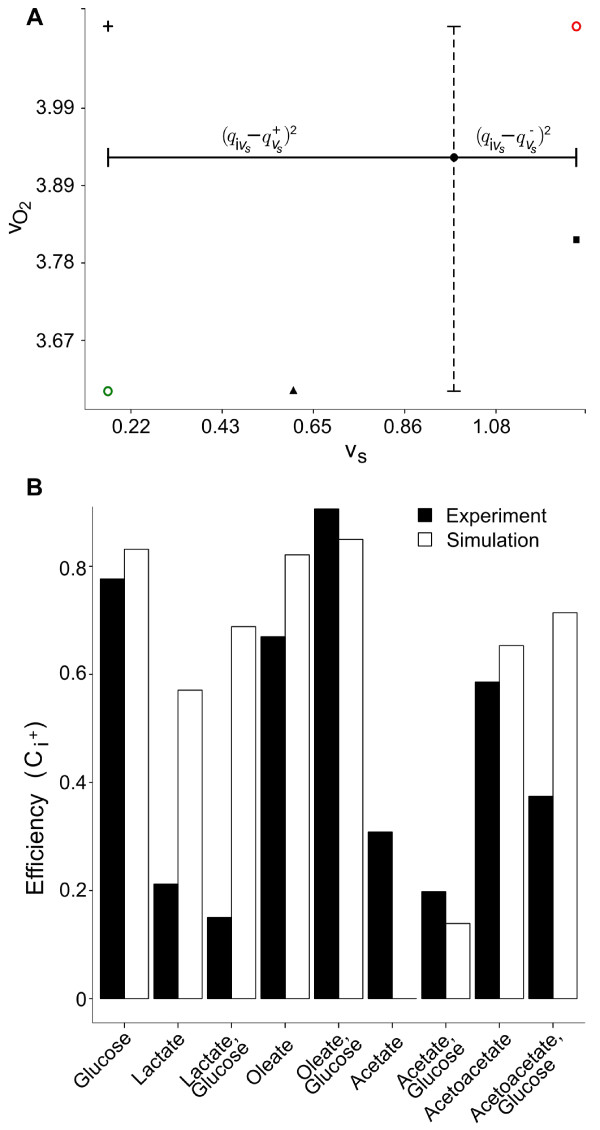
**Calculation of efficiency measure. A**. Illustration of oxygen ( $\left(v_{O_{2}}\right)$) and total substrate uptake rates (*v*_*s*_) for exclusive utilization of glucose (▴), oleate (+), acetoacetate (∙) and lactate (◼). Calculated distances for each criterion were visualized for acetoacetate, with dotted lines indicating distances to minimal and maximal oxygen uptake rate and straight lines to respective minimal and maximal total substrate uptake rates. Additional markers indicate theoretical values for best (green circle) and worst (red circle) solution. The efficiency index $\left(C_{i^{+}}\right)$ is defined as the relative distance for each solution to the best-case solution: $C_{i^{+}}=\frac{S_{i^{-}}}{S_{i^{+}}+S_{i^{-}}}$. **B**. Bar plots for comparison of calculated efficiency indices for simulated substrate compositions to experimental results [70].

The demanded ATP consumption rate could be achieved with each simulated substrate composition for glucose, oleate, acetoacetate and lactate. Nonetheless oxygen and substrate demands differed clearly between substrate combinations as presented in Figure 1. A minimal oxygen requirement for all simulations was $v_{O_{2}}=$ 3.6007 mmol·min^−1^·(l cell)^−1 ^and only reached in exclusive utilization of glucose, as can be seen in Table 3 and Figure 2A. Simulating exclusive utilization of oleate resulted with an increased oxygen uptake to a maximum of $\left(v_{O_{2}}=\right)$ 4.1101 mmol·min^−1^·(l cell)^−1^, while only a minimal total substrate uptake rate was required (*v*_*s *_= 0.162 mmol·min^−1^·(l cell)^−1^). To identify optimal substrate compositions, we used the oxygen and total substrate uptake rate as criteria. As illustrated in Figure 2A, we calculated for each simulation a euclidean based measure ( $C_{i^{+}}$,see Methods) and evaluated the difference between actual oxygen and total substrate demand to the best and worst achieved values. The optimal substrate composition should satisfy the metabolic target function, while requiring as little oxygen and substrates as possible.

**Table 3:** Simulation of varied substrate availability: baseline ATP consumption

***β***_**Glucose**_	***β***_**Oleate**_	***β***_**Acetoacetate**_	***β***_**Lactate**_	$\left(v_{O_{2}}\right)$	***v***_***s***_	$\left(C_{i^{+}}\right)$
				$\left[\frac{\text{mmol}}{\text{min}\cdot (l\;\text{cell})}\right]$	$\left[\frac{\text{mmol}}{\text{min}\cdot (l\;\text{cell})}\right]$	
0.00	1.00	0.00	0.00	4.1101	0.1626	0.6849
1.00	0.00	0.00	0.00	3.6007	0.6005	0.6576
0.00	0.00	1.00	0.00	3.9269	0.9806	0.2791
0.00	0.00	0.00	1.00	3.8124	1.2701	0.2091
0.79	0.21	0.00	0.00	3.8534	0.3823	0.7334
0.42	0.37	0.09	0.12	3.9830	0.3154	0.7000
0.33	0.19	0.43	0.05	3.9420	0.4536	0.6500
0.20	0.16	0.26	0.38	3.9485	0.5292	0.6000
0.46	0.03	0.46	0.05	3.7757	0.6847	0.5500
0.17	0.09	0.01	0.73	3.8880	0.7020	0.5000
0.20	0.04	0.41	0.35	3.8534	0.7841	0.4500
0.16	0.03	0.33	0.48	3.8448	0.8575	0.4000
0.06	0.05	0.03	0.86	3.8794	0.8942	0.3600
0.02	0.02	0.52	0.44	3.9010	0.9677	0.3000
0.07	0.00	0.07	0.86	3.7930	1.1556	0.2500
0.00	0.00	0.13	0.87	3.8297	1.2247	0.2070

All data is ranked by the calculated efficiency in descending order and given fully for exclusive utilization of each substrate. Results for altered substrate availability are listed by the achieved efficiency with a value of: 0.25, 0.3,0.36, 0.4, 0.45, 0.5, 0.55, 0.6, 0.65 and 0.7. A substrate combination of 79% glucose and 21% oleate to the total substrate uptake rate attained the highest calculated efficiency ( $\left(C_{i^{+}}=0.7334\right)$). Least efficient ( $\left(C_{i^{+}}=0.2070\right)$) was a combination of 13% acetoacetate and 87% lactate. For a complete overview of all results, please see Additional file 8 and 9. $\left(v_{O_{2}}\right)$ indicates oxygen demand; v_s_, total substrate uptake rate; and $\left(C_{i^{+}}\right)$, calculated efficiency.

In the extreme situation that only one substrate is exclusively oxidized, glucose and oleate were almost identical optimal in terms of oxygen and total substrate demand according to our selected efficiency measure ( $\left(C_{i^{+}}\right)$; Table 3, Figure 2A). Simulating more physiological situations in which all four substrates could be utilized, we determined an oleate and glucose percentage of 21% and 79%, respectively, most optimal ( $\left(C_{i^{+}}\right)$ = 0.7334; see Table 3). In contrast predominate utilization of lactate showed to be least optimal. Especially sole lactate utilization required a maximal total substrate uptake rate of v_s _= 1.2701 mmol·min^−1^·(l cell)^−1^, thus 7.84-fold higher than in case of oleate utilization. We determined the lowest efficiency for a total substrate combination of 13% acetoacetate and 87% lactate ( $\left(C_{i^{+}}=0.2070\right)$, see Table 3).

To identify alternate flux solutions that can equally satisfy the problem, i.e. yield the same optimal solution, we performed additional simulations (see Methods). The optimization problem was repeated for substrate combinations with the highest and lowest calculated efficiency (see Table 3). We determined 4 alternative distributions yielding the same optimal solution for the substrate combination achieving the highest calculated efficency (79% oleate, 21% glucose). The calculated distributions showed no significant difference from the original distribution (F = 135, Pr(> F) < 2e-16, p ≤ 0.001) and 90.70% of the fluxes were unique, thus the variance (s) equal to zero (see Additional file 10).

Furthermore, 11 alternative distributions were identified for the least optimal substrate combination showing no significant difference from the original distribution (F = 135, Pr(> F) < 2e-16, p ≤ 0.001) and 74.41% unique flux solutions (s = 0). The largest variance (s = 0.24) was found for flux rates of creatine and phosphocreatine transport into the mitochondrion as well as cytosolic and mitochondrial creatine kinase. However, we found all fluxes representing the external substrate and oxygen uptake with unique solutions (see Additional file 10).

### Validation of calculated efficiency

To validate our results, we simulated substrate compositions as determined in a recent experimental study [70], which investigated the utilization of glucose, lactate, oleate, acetate and ketone bodies (acetoacetate, (R)-3-hydroxybutanoate) in dependence of workload and insulin to improve the perfusion system for the isolated rat heart. We performed simulations with substrate compositions as present in the experiments and determined flux distributions while assuming again a baseline ATP consumption rate (v_ATPase_) of 21.6 mmol·min^−1^·(l cell)^−1^[69]. The results, summarized in Table 4 and Figure 2B, show that calculated flux rates are in good concordance with experimentally determined uptake rates and correspond in many cases. The oxygen demand is underestimated in all simulations compared to the experiment but in sole acetate oxidation.

**Table 4:** **Simulation of experimental substrate supply and comparison by calculated efficiency**$C_{i^{+}}$

				**Simulation**					**Experiment**			
**Exp.**	**Substrate**	***β***_***m***_		$\left(v_{O_{2}}\right)$	***v***_***s***_	$\left(v_{O_{2}}/v_{s}\right)$	$\left(C_{i^{+}}\right)$		$\left(v_{O_{2}}\right)$	***v***_***s***_	$\left(v_{O_{2}}/v_{s}\right)$	$\left(C_{i^{+}}\right)$
				$\left[\frac{\text{mmol}}{\text{min}\cdot (l\;\text{cell})}\right]$	$\left[\frac{\text{mmol}}{\text{min}\cdot (l\;\text{cell})}\right]$				$\left[\frac{\text{mmol}}{\text{min}}\right]$	$\left[\frac{\text{mmol}}{\text{min}}\right]$		
1	Glucose	1.00		3.60	0.60	6.00	0.83		4.28	0.55	7.84	0.78
2.1	Lactate	1.00		3.81	1.17	3.25	0.60		4.71	1.41	3.34	0.21
2.2	Glucose	0.23		3.73	0.96	3.89	0.69		4.70	1.59	2.96	0.15
	Lactate	0.77										
3.1	Oleate	1.00		4.11	0.16	25.37	0.82		4.94	0.18	28.07	0.67
3.2	Glucose	0.54		4.02	0.24	16.75	0.85		4.36	0.21	20.57	0.91
	Oleate	0.46										
4.1	Acetate	1.00		4.8	2.4	2	0		4.28	1.68	2.55	0.31
4.2	Glucose	0.05		4.6	2.11	2.19	0.14		4.69	1.46	3.21	0.2
	Acetate	0.95										
5.1	Acetoacetate	1.00		3.93	0.98	4.00	0.65		4.20	0.53	7.89	0.59
5.2	Glucose	0.26		3.81	0.86	4.41	0.71		4.95	1.01	4.91	0.37
	Acetoacetate	0.74										

$\left(v_{O_{2}}\right)$ indicates oxygen demand;v_s_, total substrate uptake rate; $\left(C_{i^{+}}\right)$, calculated efficiency.

Moreover, the total substrate uptake rate is increased in simulations for sole utilization of acetoacetate and in combined utilization of acetate and glucose. Here, the ratio of calculated oxygen demand to total substrate uptake rate shows the greatest deviance to experimentally obtained values. To further compare our simulations with the experiment, we determined for each substrate composition efficiency measures as described above. As depicted in Figure 2B, the calculated efficiency indices were almost identical except for simulations of lactate oxidation. Here, the required oxygen and substrate demand to satisfy the baseline ATP consumption rate obtained a more favourable relation as the calculated oxygen demand in sole oleate utization was clearly underestimated. Furthermore, the oxygen and substrate demand increased to a maximum ( $\left(C_{i^{+}}=0\right)$, see Table 4, Figure 2B) in simulations of sole acetate utilization. This explains differences between calculated efficiency indices. In agreement with our simulations, oxidation of glucose and oleate showed to be more optimal in terms of oxygen demand and total substrate uptake rate.

The comparison of experimental results in oxidation of acetoacetate with our simulations are limited, due to reduced cardiac work during the perfusion experiment and altered substrate application. Our simulations consider direct presence of acetoacetate and glucose, while in the experiment glucose was added at a later time in the perfusion. The data, summarized in Table 4, shows that sole utilization of acetoacetate is less efficient than glucose or oleate oxidation, but seems to achieve more favourable oxygen and substrate uptake rates than acetate and lactate.

### Calculation of substrate and oxygen uptake rates for satisfying a cardiomyocyte target function in varied substrate availability

Cardiomyocytes have to maintain an adequate ATP synthesis together with a multitude of metabolic functions including abundance of contractile proteins, membrane integrity and protection against reactive oxygen species. To reflect these metabolic functions, we extended the metabolic target function (*v*_*t*_) by including the production of NADPH besides ATP and important membrane lipids: ceramide (cer), cardiolipin (cl), phosphatidylcholine (pc), phosphatidylethanolamine (pe) and sphingomyelin (sm).

(3) $$\begin{array}{ll} v_{t}=v_{\text{ATPase}}+v_{\mathrm{cer}}+v_{\mathrm{cl}}+v_{\mathrm{pc}}+v_{\mathrm{pe}}+v_{\mathrm{sm}} & \; \end{array}$$

The corresponding metabolic flux rates were obtained from experimentally determined synthesis rates of membrane lipids from tracer studies [71,72]. We demanded for each phospholipid species specific flux rates with respect to reported fatty acid composition of membrane lipids from human heart tissue [73,74]. All experimental flux rates were referred to a single cardiomyocyte cell volume of 2.16e-11 l (see Methods, Additional file 7). Taking into account possible short-term storage of energy, we allowed the synthesis of glycogen during simulations while restricting the glycogenolysis to a maximal rate as determined in previous investigations [75].

The myocardial defending mechanisms against hypoxia are mostly represented by NADPH to maintain reduced glutathione. To consider this aspect, we performed an initial simulation to determine the basal NADPH formation in case of unrestricted substrate supply. We determined a rate of 2.13e-05 mmol·min^−1^·(l cell)^−1^ and 3.05e-03 mmol·min^−1^·(l cell)^−1^for the cytosolic and microsomal glucose-6-phosphate dehydrogenase, while no flux was found for the NADPH producing isocitrate dehydrogenase. The rate of cytosolic glucose-6-phosphate dehydrogenase corresponded to 0.08% of the hexokinase flux rate distribution. We found an overall NADPH production rate of 1.42e-05 mmol·min^−1^·(l cell)^−1^ which was included into the simulations as a minimal required rate of NADPH synthesis in addition to the metabolic target function.

In total we performed 218618 simulations of the altered supply of 9 different substrates (ns=9). As expected, no solutions were found in case of absent alpha-linoleate and docosahexaenoate supply, thus these fatty acids are required as a precursor for the biosynthesis of certain phospholipid species in the metabolic network and cannot be replaced by any other substrate [76].

**Figure 3:**
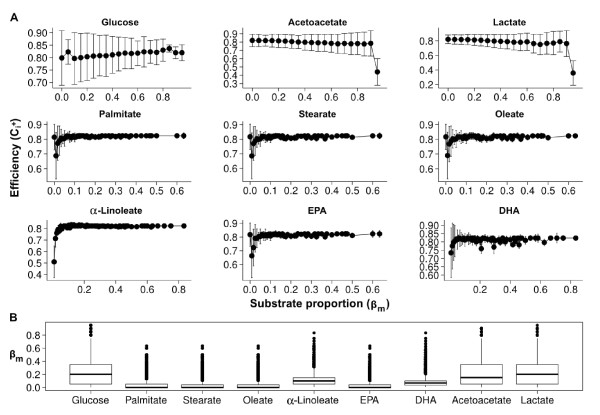
**Calculation of substrate and oxygen uptake rates in varied substrate availability. A**. Calculated efficency values for each substrate in varied share (*β*_*m*_) of the total substrate uptake rate (*v*_*t*_). Values are mean ± standard deviation. **B**. Box and whisker plots of the proportion of substrates according to the total substrate uptake flux by efficiency score $\left(C_{i^{+}}\right)$ greater than 0.8. The bold horizontal line in panels indicate mean. $\left(C_{i^{+}}\geq 0.8\right)$: n = 76925. ° Outliers.

To identify optimal substrate combinations, we calculated for each simulation the efficiency index ( $\left(C_{i^{+}}\right)$) based on three criteria: (i) oxygen demand ( $\left(v_{O_{2}}\right)$), (ii) total substrate uptake rate (*v*_*s*_) and (iii) endogenous glucose derived from glycogen turnover (*v*_*GL*_). We identified high efficiency indices for substrate combinations with a major share of fatty acids and glucose, as presented in Table 5. Especially a substrate combination of 90% Glucose, 5% of palmitate and 1.667% of alpha-linoleate, eicosapentaenoate and docosahexaenoate showed to be more favourable than any other substrate combination ( $\left(v_{O_{2}}=6.9154\;\text{mmol}\cdot {\text{min}}^{-1}\cdot {\left(l\;\text{cell}\right)}^{-1}\right)$; *v*_*s *_= 4.8859 mmol·min^−1^·(l cell)^−1^ ; $\left(C_{i^{+}}\right)$ = 0.8438). As expected, the efficiency ( $\left(C_{i^{+}}\right)$) were directly proportional to increasing share of glucose and fatty acids (Figure 3A) and inversely proportional increasing share of acetoacetate and lactate (Figure 3A). In fact, a substrate combination of 95% acetoacetate and 5% fatty acids with a share of 0.83% palmitate, 0.83% alpha-linoleate and 3.33% docosahexaenoate resulted to be least optimal to fulfil the demanded metabolic target function (see Table 5). The oxygen demand achieved a maximum ( $\left(v_{O_{2}}=48.421\;\text{mmol}\cdot {\text{min}}^{-1}\cdot {\left(l\;\text{cell}\right)}^{-1}\right)$) in predominately utilization of acetoacetate (95%) supplemented by oleate (0.83%), alpha-linoleate (0.83%) and docosahexaenoate (3.33%), while a combination of acetoacetate (45%), lactate (35%), glucose (15%), oleate (1.67%), alpha-linoleate (0.8%) and docosahexaenoate (2.5%) required a maximal total substrate supply (*v*_*s *_= 10.2332 mmol·min^−1^·(l cell)^−1^, see Additional file 11 and 12).

**Table 5:** Simulations of substrate availability: cardiomyocyte target function

***β***_**Glucose**_	***β***_**Palmitate**_	***β***_**Stearate**_	***β***_**Oleate**_	***β***_**α−Linoleate**_	***β***_**EPA**_	***β***_**DHA**_	***β***_**Acetoacetate**_	***β***_**Lactate**_	$\left(v_{O_{2}}\right)$	***v***_***s***_	***v***_***GL***_	***v***_***GS***_	$\left(C_{i^{+}}\right)$
									$\left[\frac{\text{mmol}}{\text{min}\cdot (\mathrm{lcell})}\right]$	$\left[\frac{\text{mmol}}{\text{min}\cdot (\mathrm{l cell})}\right]$	$\left[\frac{\text{mmol}}{\text{min}\cdot (\mathrm{l cell})}\right]$	$\left[\frac{\text{mmol}}{\text{min}\cdot (\mathrm{l cell})}\right]$	
0.9000	0.0500	0.0000	0.0000	0.0167	0.0167	0.0167	0.0000	0.0000	6.9154	4.8859	0.0000	0.4027	0.8438
0.2500	0.0000	0.0000	0.0000	0.2083	0.0000	0.0417	0.0000	0.5000	4.0502	0.4093	0.1450	0.0000	0.8000
0.4500	0.0417	0.0000	0.0000	0.0417	0.0000	0.1667	0.3000	0.0000	11.4761	2.0466	0.0000	0.0000	0.7500
0.2500	0.0250	0.0000	0.0500	0.0250	0.0250	0.0250	0.5500	0.0500	14.9112	3.2573	0.0000	0.0690	0.7000
0.0000	0.0250	0.0000	0.0250	0.0250	0.0000	0.0750	0.1000	0.7500	17.3465	3.4111	0.0098	0.0000	0.6500
0.2500	0.0167	0.0167	0.0000	0.0167	0.0167	0.0333	0.6500	0.0000	20.2825	4.8859	0.0000	0.1150	0.6000
0.0500	0.0000	0.0333	0.0000	0.0167	0.0333	0.0167	0.4500	0.4000	22.5428	4.8859	0.0000	0.0103	0.5500
0.1500	0.0000	0.2250	0.0750	0.0750	0.0000	0.0750	0.1500	0.2500	25.2571	10.2332	0.0000	0.4532	0.5000
0.4000	0.0000	0.0167	0.0000	0.0083	0.0083	0.0167	0.3500	0.2000	27.6323	9.7718	0.0000	0.3434	0.4500
0.3000	0.0000	0.0000	0.0083	0.0083	0.0000	0.0333	0.0500	0.6000	29.8822	10.2332	0.0000	0.2629	0.4000
0.2500	0.0167	0.0083	0.0000	0.0083	0.0000	0.0167	0.3000	0.4000	32.3227	10.2332	0.0000	0.2266	0.3500
0.2500	0.0083	0.0083	0.0000	0.0083	0.0000	0.0250	0.5000	0.2000	34.8810	10.2332	0.0000	0.2266	0.3000
0.2000	0.0000	0.0250	0.0000	0.0083	0.0083	0.0083	0.7500	0.0000	37.2907	9.7718	0.0000	0.1757	0.2500
0.0000	0.0000	0.0083	0.0083	0.0083	0.0083	0.0167	0.3000	0.6500	38.8292	9.7718	0.2160	0.0000	0.2000
0.0000	0.0083	0.0083	0.0083	0.0083	0.0000	0.0167	0.4000	0.5500	41.2341	10.2332	0.2160	0.0000	0.1500
0.0000	0.0167	0.0000	0.0000	0.0083	0.0167	0.0083	0.9500	0.0000	44.3334	9.7718	0.0000	0.0029	0.1000
0.0000	0.0000	0.0083	0.0000	0.0083	0.0000	0.0333	0.9000	0.0500	47.5569	10.2332	0.0120	0.0000	0.0500
0.0000	0.0083	0.0000	0.0000	0.0083	0.0000	0.0333	0.9500	0.0000	47.9063	10.2332	0.2160	0.0000	0.0100

Results are shown for simulations of varied substrate availability of glucose, acetoactate, lactate and 6 different fatty aicids: palmitate, stearate, oleate, alpha-linoleate, eicosapentaenoate and docosahexaenoate. For each simulated composition efficiency values $\left(C_{i^{+}}\right)$ were calculated based on three criteria q_j_ (j=1,2,..,nj): (1) oxygen requirement ( $\left(v_{O_{2}}\right)$

Again we repeated the optimization problem to identify alternative flux solutions (see Methods) in substrate combinations with the highest and lowest calculated efficiency (see Table 5). Here, we identified 202 alternative distributions with 71.92% unique flux solutions (variance s=0) achieving the same objective for the substrate combination with the highest calculated efficiency (see Additional file 10). The alternative distributions were without significant difference from the original distribution (F = 462, Pr(> F) < 2e-16, p ≤ 0.001).

Similar, 216 alternative distributions with 56.96% unique flux solutions were found for the substrate combination with lowest calculated efficiency. The calculated distributions showed no significant difference from the original distribution (F = 278, Pr(> F) < 2e-16, p ≤ 0.001). The largest variance (s = 29.83) was again found for flux rates of creatine and phosphocreatine transport into the mitochondrion as well as cytosolic and mitochondrial creatine kinase. In addition we found variability for beta-oxidation of fatty acids and ATP:nucleoside-diphoshate phosphotransferase. Each flux representing the glycogenolysis, external substrate and oxygen uptake was found unique (s=0) in all simulations (see Additional file 10).

**Figure 4:**
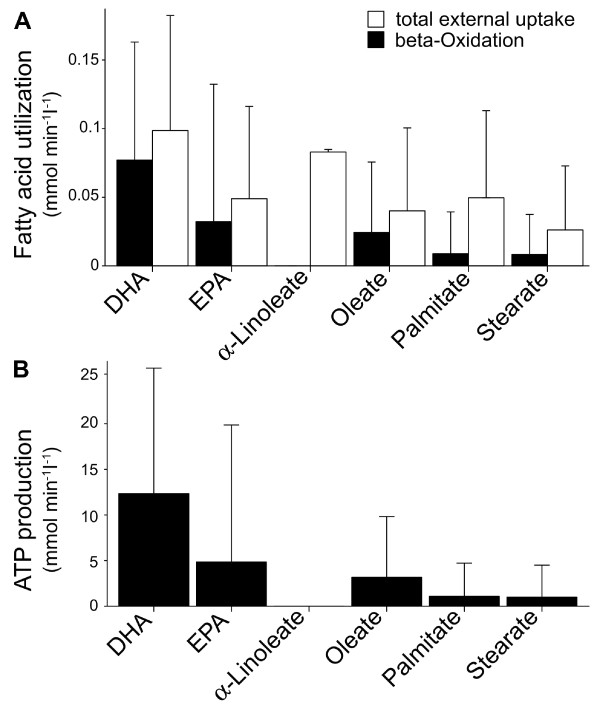
**Exogenous utilization of fatty acids and ATP production. A**. Exogenous utilization of fatty acids and contribution to ATP production by fatty acid beta-oxidation. **B**. Steady state rates of ATP production from fatty acid beta-oxidation. Data are expressed as boxplots.

The data, as illustrated in Figure 3B, shows that the variability of substrate combinations with a large efficiency index ( $\left(C_{i^{+}}\geq \right)$0.8) increased with the advanced objective function. The mean share ( $\left(\beta_{\bar{x}}\right)$) of glucose, fatty acids, acetoactetate and lactate where 0.231, 0.311, 0.221, and 0.237, respectively. The data, summarized in Figure 3A, show for all fatty acids a similar pattern of calculated efficiency. Nonetheless, the contribution to ATP production differed between saturated, monounsaturated and polyunsaturated fatty acids. The mean rates for fatty acid utilization and rates of ATP produced by fatty acid utilization are summarized in Figure 4. Rates of ATP produced by beta-oxidation were calculated by assuming 120, 136, 134, 132, 142 and 156 moles of ATP derived from full oxidation of palmitate, stearate, oleate, alpha-linoleate, eicosapentaenoate and docosahexaenoate, respectively. The vast majority of palmitate, stearate and oleate contributed to phospholipid biosynthesis, while polyunsaturated fatty acids were mostly degraded via beta-oxidation. As illustrated in Figure 4A, alpha-linoleate was not degraded by beta-oxidation, thus contributed fully to phospholipid biosynthesis. In addition, we found maximal ATP production through beta-oxidation in degradation of docosahexaenoate and eicosapentaenoate (Figure 4B). In fact, up to 99.1% and 98.82% of utilized docosahexaenoate and eicosapentaenoate could contribute to ATP production, respectively.

**Figure 5:**
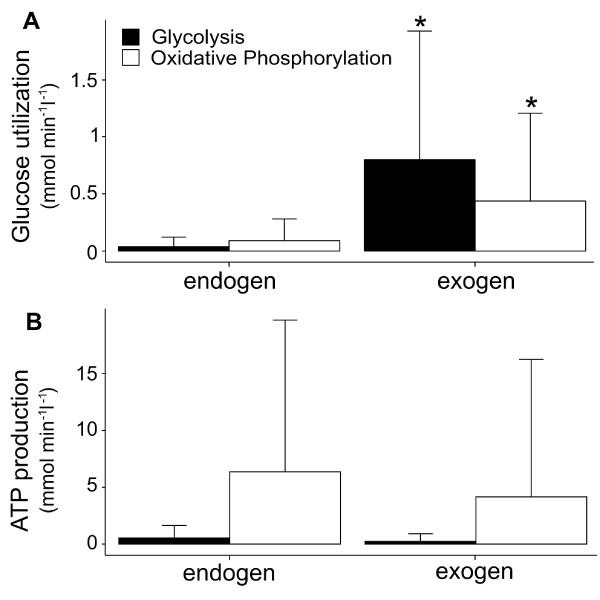
**Glucose utilization and ATP production. A**. Degradation of exogenous and endogenous glucose (glycogenolysis) by glycolysis and oxidative phosphorylation. **B**. Steady state rates of aerobic and anaerobic ATP production from glucose utilization derived from exogenous glucose and glycogenolysis. Data are expressed as boxplots. ^∗^significant difference between endogenous and exogenous glucose. p < 0.05 (unpaired t-test).

Glycogenolysis and glycogen synthesis occurred simultaneously throughout the simulations. In case of excessive glucose supply, which was not needed to fulfill the target function, glycogen was synthesized with a maximal rate of 0.887 mmol·min^−1^·(l cell)^−1^. Rates of exogenous glucose entering glycolysis were significantly greater than those from endogenous glycogen degradation (Figure 5A; p ≤ 0.05). Following this, a large proportion of exogenous glucose was oxidized by oxidative phosphorylation and was also significantly greater than those from endogenous glycogen degradation (*v*_*exo *_= 0.437 ± 0.766 mmol·min^−1^·(l cell)^−1^; *v*_*endo *_= 0.091 ± 0.189 mmol·min^−1^·(l cell)^−1^; p ≤ 0.05). In fact oxidative phosphorylation contributed the most to cellular ATP production from exogenous and endogenous glucose utilization (Figure 5B), with glycogen accounting for 34.41% of ATP production (when glucose accounted for 50% of the total substrate uptake rate). The percentage of ATP production deriving from endogenous glucose oxidation by oxidative phosphorylation increased to 80.16% in a glucose share less than 10% of the total substrate uptake rate. These results are supported by previous findings [77] showing a contribution of glycogen to 41% of the total ATP production under experimental conditions. In summary, the present results demonstrate that an optimal metabolic and physiological function of the cardiomyocyte is provided by utilization of long-chain unsaturated fatty acids, supplemented by saturated fatty acids polyunsaturated fatty acids, and exogenous glucose.

## Discussion

In this study, we developed a comprehensive reconstruction of a system-scale stoichiometric model of a human cardiomyocyte that accomplish a large set of metabolic and physiological functions to study the metabolism of cardiomyocytes. The model is based on previous human network reconstructions [2-4] and a comprehensive integration of gene expression and further available experimental evidence for metabolic reactions reported for cardiomyocytes. Using flux-balance analysis we ensured the capability of the network to achieve a wide range of metabolic target reactions required for maintaining the structural and functional integrity of the cell [5,78]. The consistency and functionality of CardioNet is a clear advantage compared to a previous automatisied genome-scale network reconstruction of the human heart [25] which lacks functionality.

CardioNet considers additional 228 mitochondrial reactions compared to two previously reported mitochondrial networks of the human cardiomyocyte [23,24]. Especially, the metabolism of 26 distinct fatty acids is included to a greater extent with consideration of variable acyl-chain composition of important phospholipids such as cardiolipin, phosphatidylserine and phosphatidylcholine. The present reconstruction may serve as a reliable basis for the integration and analysis of different types of data to study important metabolic processes of the human cardiomyocyte. The estimation of flux rates from tracer kinetic data [79,80] or prediction of flux changes inferred from changes in gene expression level of metabolic enzymes [81] under pathological conditions are only a few examples of possible applications.

Under physiological conditions cardiomyocytes are metabolizing a wide range of substrates including fatty acids, glucose, lactate, pyruvate, ketone bodies and amino acids, to meet the ATP demand for muscle contraction and further cellular mechanisms [9]. The rate of substrate utilization is dependent upon (i) substrate availability, (ii) requirement of ATP production for maintenance of cardiac contraction, (iii) oxygen supply and (iv) hormonal level of various hormones directly influencing substrate uptake, e.g. insulin. Recent investigations in other cells showed the importance of maximization of molar yield of metabolites in order to maintain cellular integrity under varied extracellular conditions [82]. In our study, we took up this point and questioned how variations in the relative proportions of glucose, lactate, fatty acids and ketone bodies may influence the efficiency of cardiac metabolism. To address this question, we applied the principle of flux minimization [5] to enable the formation of defined metabolic targets while utilizing substrates in varied proportions. By calculating a euclidean based-distance measure, we were able to identify optimal substrate combinations to maintain cardiac contraction based on the criteria: (i) oxygen demand, (ii) total substrate uptake rate and (iii) rate of endogenous glucose derived from glycogenolysis.

As shown in the present study, a predominant oxidation of fatty acids (79%) supplemented by glucose (21%) showed to maintain most efficiently the required ATP production. We found that in sole oleate utilization total substrate requirement and oxygen consumption are more favourable compared to glucose, lactate and acetoacetate. Furthermore, predominant utilization of lactate and acetoacetate was least optimal to maintain ATP production. These findings are supported by previous studies [70,83] documenting reduced cardiac performances in predominately supply of ketone bodies. In diabetic conditions with increased concentration of ketone bodies cardiac activity improved with additional fatty acid supply indicating the inadequacy of ketone bodies to efficiently maintain ATP production.

To further validate our results, we simulated substrate proportions as present in a previous study of the isolated working rat heart [70] and compared the calculated efficiency measures derived from experimental values with our simulations. We found the same ranking for efficiency of utilized substrates during simulations as in the experimental study. During the experiment, cardiac performance declined in a sole ketogenic environment (acetoacetate, (R)-3-hydroxybutanoate) while a mixture of glucose and acetoactetate seemed to reverse this effect. We calculated similar efficiency measures for ketone body utilization during simulations and with experimental values, supporting these previous findings. In addition, acetate showed the worst relation of oxygen demand to total substrate requirement to fulfil the metabolic target, both in results from simulations and experimental values. Nonetheless, no decline in cardiac performance during the actual experiment has been reported. In contrast to our results, lactate were less efficient during the experimental setting as our simulations would have suggested. These differences may be caused by substrate interactions which cannot be considered by FBA simulations, the reduced metabolic target function to ATP production without consideration of any other metabolic function and possibly observational error in the experiment.

On the basis of these results, we expanded the metabolic target function by demanding besides ATP the production of NADPH and the important membrane lipids: ceramide, cardiolipin, phosphatidylcholine, phosphatidylethanolamine and sphingomyelin. For this purpose, we further modified the fraction of fatty acids in the set of importable substrates by saturated (palmitate, stearate), monounsaturated (oleate), long chain poly-unsaturated omega-6 (alpha-linoleate) and omega-3 (eicosapentaenoic acid, doxosahexanoic acid) fatty acids. Although long chain poly-unsaturated fatty acids (PUFA) predominately serve as membrane lipids [12,15,16], there is evidence for occurrence of Acyl-CoA dehydrogenase 9 (ACAD-9) in human cardiomyocytes [84]. ACAD-9 catalyses the initial step of mitochondrial fatty acid beta-oxidation. Moreover, a previous study [85] could show enzymatic activity for ACAD-9 with long-chain unsaturated acyl-CoA as substrate (e.g.:C22:6-CoA). This is in concordance with another study [86] measuring rates of fatty acid beta-oxidation for palmitate and docosahexaenoate. Hence, it is reasonable to consider fatty acid beta-oxidation of PUFA for our simulations.

Demanding metabolic flux rates as reported in previous experimental studies of membrane lipids [71,72] and integrating the fatty acid composition of phospholipids as reported by investigations in human heart tissue [73,74], assured a physiological simulation of cardiomyocyte metabolism. We further related each flux rate to the cellular volume of a single cardiomyocyte. The myocardial defence mechanisms against hypoxia are mostly effected by NADPH to maintain reduced glutathione. To consider this aspect, we performed an initial simulation to determine the basal NADPH formation by the glucose-6-phosphate dehydrogenase, the rate limiting enzyme of oxidative pentose phosphate pathway, in case of unrestricted substrate supply. This estimated basal rate of NADPH production ( 1.42e-05 mmol·min^−1^·(l cell)^−1^) was demanded as minimal requirement in order to maintain cellular protection against ROS.

In addition, we considered cardiac short-term storage of energy in particular glycogen, which could act as a potential precursor for sn-glycerol, a known intermediate for phospholipid biosynthesis, and ATP production. During simulations we allowed the synthesis of glycogen while including the limited amount of glycogen storage by restricting glycogenolysis to a maximal rate as determined in previous investigations [75]. As shown, in the present study glycogen synthesis and glycogenolysis occurred simultaneously throughout the simulations, which is well in accordance with previous studies [75,77] documenting the same pattern. In case the available glucose was not needed to fulfil the metabolic target function, glycogen was synthesized with a maximal rate of 0.887 mmol·min^−1^·(l cell)^−1^. The vast majority of utilized glucose during simulations derived from exogenous uptake, indicating that endogenous glycogenolysis was only utilized in a decreased glucose supply. Both external and endogenous glucose essentially contributed to ATP production by oxidative phosphorylation.

Previously, Henning et. al. [77] demonstrated that glycogen accounted for 41% of synthesized ATP in predominately glucose oxidation. In agreement with this study, our results show a glycogen contribution to ATP synthesis from glucose oxidation which is dependent on exogenous glucose supply. In case glucose accounts for 50% of the total substrate uptake rate, we found 34.41% of ATP production from oxidative phosphorylation is related to glycogen. Consequently with decreased glucose supply to 25% of the total substrate uptake rate, we found an increased contribution of glycogen up to 80.16%.

In order to test the robustness of our solutions we analysed the uniqueness of calculated flux solutions. Depending on the complexity of the target function we found up to 90.70% fluxes with unique solutions which included flux rates for glycogenolysis and external substrate and oxygen uptake for every tested substrate combination. Although a certain amount of flux solutions showed variability none of the alternative distributions showed significant difference from the original distribution. The efficency analysis of substrate combinations is not compromised by this small varibility and based on fluxes with unique solutions.

To our knowledge this is the first study investigating the efficiency of a large set of substrates, including long-chain fatty acids, through simulation. We found utilization of substrate combinations with a mixture of all investigated substrate more efficient compared to sole utilization of single substrates. Here predominant utilization of fatty acids ( $\left(\beta_{\bar{m}}\right)$ = 0.311), especially long-chain unsaturated fatty acids, supplemented by glucose ( $\beta_{\bar{m}}$ = 0.231), acetoacetate ( $\beta_{\bar{m}}$ = 0.221) and lactate ( $\beta_{\bar{m}}$ = 0.237) seemed to be more favourable with the extended metabolic target function which is in good concordance with our previous findings. Moreover, utilization of predominantly saturated and C18 polyunsaturated fatty acids seemed to be more favourable, than a greater share of long chain omega-3 poly-unsaturated fatty acids. This is supported by a recent study [87] in isolated muscle fibres from diabetic hearts, where an increase of mitochondrial uncoupling was measured during exposure to fatty acid. The induced ROS production in cardiomyocyte mitochondria led to activation of multiple adaptive mechanisms by which oxidative damage can be prevented. The ambivalent role of long chain omega-3 poly-unsaturated fatty acids is supported by findings from other studies [15,16], that showed beneficial metabolic effects in myocardial protection against oxidative damage by incorporation of long chain omega-3 poly-unsaturated fatty acids into mitochondrial membrane phospholipids such as cardiolipin.

Our results indicate, that a switch from fatty acid utilization to predominant glucose utilization in situations with increased workload would not decrease the efficiency of cardiac metabolism. This is indicated by a efficiency value ( $\left(C_{i^{+}}\right)$ = 0.8438) for a combination of 90% glucose, 5% palmitate, 1.67% alpha-linoleate, 1.67% eicosapentaenoate and 1.67% docosahexaenoate. During the simulations polyunsaturated fatty acids contributed most to ATP production via beta-oxidation, while the vast majority of palmitate, stearate and oleate were used for phospholipid biosynthesis. In fact, up to 99.1% of utilized docosahexaenoate and 98.82% of utilized eicosapentaenoate could contribute to ATP production through degradation during beta-oxidation, respectively. In addition, we found alpha-linoleate did not undergo beta-oxidation, but served as substrate in the biosynthesis of phospholipid. Consistent with our previous findings, we found predominantly utilization of acetoacetate least optimal with the extended metabolic target function. Here, the oxygen demand and mitochondrial oxygen consumption were greater than with any other substrate combination, thus, possibly, increasing the flux through complex I of the mitochondrial oxidative phosphorylation which is considered a main source of cellular ROS. Our observations suggest that under metabolic conditions with increased acetoacetate levels (e.g. diabetes) cardiac contractility would be affected by decreased ATP formation and increased ROS formation, which is linked to mitochondrial dysfunction [18,19]. Nonetheless, it is beyond the scope of this study to further analyse these mechanisms but our network reconstruction might contribute to further investigation by incorporation of gene expression level information of metabolic enzymes mediated by PPAR.

## Conclusions

In summary, our study provides a comprehensive, reconstruction of the metabolic network of the human cardiomyocyte (CardioNet) to study metabolic and physiological functions of the cardiomyocyte.

The evaluation of metabolic efficiency in substrate supply and utilization necessitates consideration of oxygen and substrate demand, as well as endogenous glucose deriving from glycogenolysis. In aerobic conditions predominant utilization of saturated and long-chain unsaturated fatty acids supplemented by glucose proved to be more favourable for efficient cardiac metabolism than utilization of acetoacetate or lactate. Furthermore, we showed, that glycogenolysis and glycogen synthesis occurred simultaneously. In conclusion, CardioNet can serve as a reliable basis to study cardiomyocyte metabolism.

## Methods

### Metabolic network reconstruction

To identify a tissue specific set of metabolic reactions we applied the algorithm by Shlomi et al. [6], which integrates gene expression data with linear optimization problem. The approach aims to find a stationary flux distribution by maximizing the number of reactions whose activity is consistent with their gene expression state [6]. We obtained gene expression information in normal human heart tissue samples from two different datasets (GDS181 [88], GSE1145) which are available from Gene Expression Omnibus [89]. A gene was considered to be expressed if the expression value was equal or greater than a threshold value of 100 or the Affymetrix Call indicated a present expression.

By using the Ensembl Homo sapiens database [90] and KEGG orthology records [91], we mapped these Affymetrix probe set IDs to reactions of the global reconstruction of the human metabolic network - Recon1 [2]. After applying the algorithm by Shlomi et al. [6] the resulting subnetwork comprised of 972 reactions and metabolites for which corresponding reactions in KEGG were identified based on the Enzyme Commission (EC) number. We extended this initial set of reactions by including further KEGG reactions [91] associated with EC numbers that are annotated as present in heart tissue by additional databases such as the Braunschweig Enzyme database (BRENDA) [92] and UniProtKB [93]. Further information about metabolites not obtained from KEGG were integrated by using the Human Metabolome Database (HMDB) and Lipid Maps Classification System [26] (see Additional file 1).

Reactions were evaluated for tissue specificity and subcellular localisation according to database knowledge and reported experimental evidence from scientific literature. We included reactions into the network if evidence were found for occurrence in human cardiomyocytes or heart tissue in general (e.g.: heart muscle, myocard, cardiac tissue). In case no evidence were found for humans, we relied on other mammalian species and human orthologous genes allowing inference of the reaction. Information on transport reactions was obtained from the Transport Classification database [94], Reactome database [95] and another metabolic network of the human hepatocyte (HepatoNet1, [4]) providing a large set of manually curated transport processes. We included transport process from this study in case we found evidence for occurrence in human cardiomyocyte.

Each reaction in the network was assigned to one of the following sub-cellular localisation: external, cytosol, mitochondrion, lysosome, peroxisome and microsome. The compartments endoplasmic reticulum (ER), Golgi apparatus and microsome are represented in the metabolic network as one combined compartment, microsome. Communication among endoplasmic reticulum, Golgi apparatus and microsome is mediated by vesicular transport processes which can be only inadequately included into the FBA methodology. In addition, recent studies demonstrated the experimental difficulty of proteomic profiling of the microsomes [96,97]. The determination for sub-cellular localisation was based on experimental evidence (protein localisation, targeting sequences and subcellular fractionation) and indirect physiological or biochemical evidence. In the absence of information, reactions were assigned to the cytosolic compartment (see Additional file 1 and 5). The directionality of reactions were set according to Gibbs energy (ΔG) as obtained from a prediction method [98] (see Additional file 2).

For the integration of different level of information we used the METANNOGEN software [99]. The complete overview of present genes in the metabolic network is provided in Additional file 13. The final reconstructed network is available in SBML format (see Additional file 5).

#### Flux balance analysis

The network was subjected to further flux-balance simulations with different metabolic objectives to test functionality (see Additional file 3). The optimization objective has been the minimization of internal fluxes [5]. We defined a set of exchangeable metabolites which were applied as constraints in the optimization problem (see Additional file 3). In addition to this, the reconstructed network was subjected to functional pruning [78] by reducing the model to a smaller sub-network which contains no dead-ends or blocked reactions that may not carry a non-zero flux. For this purpose, we defined a set of exchangeable metabolites (see Additional file 3) used for pruning based on metabolic and physiological function of the cardiomyocyte (see Table 2). This process revealed (i) missing reactions, (ii) missing transporters and (iii) incomplete reaction directionality which were part of further curation process.

#### Calculating uptake rates of substrates and oxygen in varied substrate supply

We used the metabolic network to assess the influence of nutritional blood supply on the metabolic efficiency of the cardiomyocyte to accomplish various metabolic objectives. For this purpose, we defined the metabolic target v_t_ as a linear combination of all those fluxes *v*_*r*_(r=1,2,..,nt), which have to be accomplished by the network in order to maintain cellular integrity and cardiac contractility:

(4) $$\begin{array}{ll} v_{t}=\overset{\text{nt}}{\underset{r=1}{\sum }}v_{r}. & \; \end{array}$$

The metabolic flux rates *v*_*r *_were obtained from experimentally determined synthesis rates which were taken from previous investigations [69,71,72]. We further considered the variable fatty acid composition of phospholipids as reported in human heart tissue [73,74]. The detailed list of included synthesis rates is provided in the Additional file (see Additional file 7). To simulate altered substrate availability, we defined the total substrate uptake flux (*v*_*s*_) as a linear combination of the external uptake rates *v*_*m *_(m=1,2,..,ns) for each oxidized substrate n. The substrate availability in the external space is reflected by the coefficient *β*_*m*_ with

(5) $$\begin{array}{ll} \overset{\text{ns}}{\underset{m=1}{\sum }}\beta_{m}=1,\;0\leq \beta_{m}\leq 1. & \; \end{array}$$

The higher the share of substrate n in the external medium, the higher its share in the total substrate uptake space (*v*_*s*_). This assumption is justified by experiments carried out with the perfused isolated heart [70] showing the relation between measured uptake rates of various energy-delivering substrates directly reflected the ratio of these substrates in the perfusion medium. The resulting substrate uptake rate for each simulated substrate composition i (i=1,2,..,ni) reads, as follows:

(6) $$\begin{array}{ll} v_{m_{i}}=(\beta_{m_{i}}\cdot v_{s}). & \; \end{array}$$

The optimization problem was described for accomplishing the metabolic target flux (*v*_*t*_) while minimizing the sum of the total substrate uptake rate (*v*_*s*_) and oxygen uptake rate ( $\left(v_{O_{2}}\right)$). The internal and exchange fluxes of the metabolic network were defined as v and the stoichiometric matrix of the complete metabolic network as N. The lower and upper bounds on fluxes are expressed as *v*_*min*_ and *v*_*max*_.

The optimization problem according to the flux-minimization principle for each simulated substrate composition i reads, as follows:

(7) $$\text{minimize}\;\;\;\;\;\;(v_{s}+v_{O_{2}})$$

(8) subject toN·v=0,

(9) $$v_{\min,j}\leq v\leq v_{\max,j},$$

(10) $$v_{t}=\overset{\mathrm{nt}}{\underset{r=1}{\sum }}v_{r},$$

(11) $$v_{m}=(\beta_{m}\cdot v_{s}).$$

#### Alternate optima

Presumed the original problem is feasible and a value for the objective can be calculated, the solution for v (see equation (8)) is not necessarily unique. Multiple solutions might occur to solve the problem and cause degeneration of the flux distribution. In order to identify alternate flux solutions that can equally satisfy the problem, i.e. yield the same optimal solution, we performed additional simulations. The MILP was re-solved after adding a constraint (*z*^∗^) for a single flux of the original flux distribution which was set to either 1.01-fold ( $\left(z_{1}^{*}\right)$) or 0.99-fold ( $\left(z_{2}^{*}\right)$) of its original calculated flux value (*v*_0_). We repeated this additional constraining for one flux after the other and resolved the optimization problem.

(12) $$z_{1}^{*}=1.01\cdot v_{0},$$

(13) $$z_{2}^{*}=0.99\cdot v_{0}.$$

The modified optimization problem reads as follows:

(14) $$\text{minimize}\;\;\;\;(v_{s}+v_{O_{2}})$$

(15) subject toN·v=0,

(16) $$v_{\min,j}\leq v\leq v_{\max,j},$$

(17) $$v_{t}=\overset{\mathrm{nt}}{\underset{r=1}{\sum }}v_{r},$$

(18) $$v_{m}=(\beta_{m}\cdot v_{s})$$

(19) $$z_{1}^{*},z_{2}^{*}.$$

In case no feasible solution could be found, the respective original flux solution is dependent on one or more fixed fluxes of the target function and cannot be varied. Each feasible solution yielding the same optimum as the original was considered for further variance analysis. In case the variance is equal to zero the respective flux is uniquely defined. On the other hand, indicates a non-zero flux value an unequivocally definition of the respective flux. These fluxes may vary without affecting the optimal behaviour of the metabolic network, based on the capability of the network to compensate these variations. We repeated the optimization problem for substrate combinations which were identified with the highest or lowest efficiency value while satisfying (1) a baseline ATP consumption rate and (2) a target function of the cardiomyocyte ( see Additional file 10). Based on the variance analysis for all four examples, we found no significant difference between the solutions. About one third of the fluxes cannot be changed without violating the demanded target function, thus leading to an infeasibility of the problem.

#### Euclidean based distance measure for efficiency

To identify optimal substrate combinations within all simulations of altered substrate availability, we used as criteria *q*_*j*_ (j=1,2,..,nj): the oxygen demand ( $\left(v_{O_{2}}\right)$, *q*_1_), total substrate uptake rate (*v*_*s*_,*q*_2_) and endogenous glucose derived from glycogenolysis (*v*_*GL*_, *q*_3_). Optimal substrate combinations should not only meet a minimal distance to the best achieved solution, but also a maximal distance to the worst achieved solution for each criterion. This takes into account, that one solution could show minimal requirement of substrates while the oxygen demand and glycogenlysis increases. Euclidean distances were calculated for every simulated substrate composition i (i=1,2,..,ni) by, first, determine the best-case ( $\left(q_{j}^{+}\right)$) and worst-case solution ( $\left(q_{j}^{-}\right)$) from all simulations for each criterion as the minimal and maximal uptake rate, respectively. Secondly, the distances between actual flux rate (*q*_*ji*_) to the best-case ( $\left(q_{j}^{+}\right)$) and worst-case solution ( $\left(q_{j}^{-}\right)$) were calculated.

(20) $$S_{i^{+}}=\sqrt{\overset{\mathrm{nj}}{\underset{j=1}{\sum }}{\left(q_{\mathrm{ij}}-q_{j}^{+}\right)}^{2}}\;\;\;\forall \;i=1,\mathrm{..},\mathrm{ni},$$

(21) $$S_{i^{-}}=\sqrt{\overset{\mathrm{nj}}{\underset{j=1}{\sum }}{\left(q_{\mathrm{ij}}-q_{j}^{-}\right)}^{2}}\;\;\;\forall \;i=1,\mathrm{..},\mathrm{ni}.$$

The relative distance for each solution to the best-case solution is defined as the efficiency index $\left(C_{i^{+}}\right)$ for the considered substrate combination:

(22) $$\begin{array}{ll} C_{i^{+}} & =\frac{S_{i^{-}}}{S_{i^{+}}+S_{i^{-}}}\; \end{array}$$

with a maximal theoretical efficiency index $\left(C_{i^{+}}\right)$ equal to 1. Substrate compositions with the highest overall match and efficiency indices $\left(C_{i^{+}}\right)$ close to 1 were considered as optimal solutions for the chosen metabolic objective.

#### Cardiomyocyte volume

We referred all flux calculations to the volume of one single cardiomyocyte to integrate experimental flux rates from different studies (see Additional file 7). The cardiomyocyte volume (*V*_*myo*_) was calculated as follows: $V_{\mathrm{myo}}=\frac{\Pi }{4}\cdot d^{2}\cdot l$, with a diameter (d) of 14 μm and length (l) of 140 μm [100,101].

(23) $$\begin{array}{ll} \left(V_{\mathrm{myo}}\right) & =2.16\;e-11l.\; \end{array}$$

#### Computation

The computation was performed with the aid of CPLEX 10.1 (ILOG, Gentilly, France) and FASIMU [102].

#### Statistical analysis

Comparison of mean values between groups was evaluated with unpaired t-test. A value of p < 0.05 was considered significant. Statistical significance between flux solutions for the analysis of alternate flux solutions was determined by use of 1-way ANOVA. Vertical lines in the histograms indicate means ± SE.

## Abbreviations

FBA: Flux balance analysis; CHF: Congestive heart failure; ROS: Reactive oxygen species; EPA: Eicosapentaenoate; DHA: Docosahexaenoate; GL: Glycogenolysis; GS: Glycogen synthesis; SE: Standard error; MILP: Mixed Integer Linear Problem.

## Competing interests

The authors declare that they have no competing interests.

## Authors’ contributions

AK developed the original idea, carried out all computational analyses and drafted the manuscript. HGH, DF, HSR, GK and VRZ participated in the design and evaluation of the analyses. All authors contributed to and approved the final manuscript.

## Supplementary Material

Additional file 1 **Metabolites of the metabolic network.** Metabolites listed in this table occur in the metabolic network. For each entry a unique network identifier is given and provided with information of metabolite title, title synonym, metabolite sum formula and assigned compartment. Additionally cross-references to other databases are given and refer to the following databases: UniProtKB (UniProtKB entry), KEGG (Compound ID), Lipid Maps (LM ID), Pub Chem (CID) and Human Metabolome Database (HMDB ID). Abbreviations used in the table for compartments are as following, ext: external, cyto: cytosol, mito: mitochondrion, lyso: lysosome, peroxy: peroxisome and micro: microsome. Click here for file

Additional file 2 **References.** During the network reconstruction additional evidence for occurrence of metabolic reactions in the cardiomyocyte were obtained from previously reported studies. This table gives a full list of cross-references to PubMed identifier (PMID) providing evidence for included reactions of the metabolic network. Furthermore, directionality of reactions was set according to Gibbs energy (ΔG, kJ/mol) and is provided with this table. Click here for file

Additional file 3 **Definition and overview of objectives and constraints for simulation of metabolic and physiological functions of the cardiomyocyte.** To ensure consistency and full functionality of the metabolic network, we performed a critical testing of physiological functions based on knowledge of the cardiac metabolism by using flux balance analysis. The table lists all objectives and applied constraints as used in the optimization problem. Furthermore, constraints as used in functional pruning of the network are given. Abbreviations for constraints as used in simulations with FASIMU software are as follows: (+), secretion of the metabolite is allowed or the metabolite is product; (-), uptake of the metabolite is allowed or metabolite is substrate and (=), secretion and uptake of the metabolite is allowed or metabolites is either product or substrate. Click here for file

Additional file 4 **Flux distributions of metabolic and physiological functions of cardiomyocyte.** To ensure consistency and full functionality of the metabolic network, we performed a critical testing of physiological functions based on knowledge of the cardiac metabolism by using flux balance analysis. Flux distributions listed in this table have been predicted for each metabolic objective as defined in Additional file 5. Abbreviations for compartments: ext - external, cyto - cytosol, mito - mitochondrion, lyso - lysosome, peroxy - peroxisome, micro - microsome. Click here for file

Additional file 5 Metabolic network of the human cardiomyocyte in SBML format. Click here for file

Additional file 6 **Testing functionality of Human heart model.** A comparison of the metabolic network to a previously reported genome-scale reconstruction of the human heart [25] was performed. The presented physiological functions of the cardiomyocyte (see Additional file 13) were applied to test the functionality of the partial network of the human heart and compare the performance of both networks. From 110 tested functions 53 were found to have no feasible solution, this included important cellular functions such as the citric acid cycle. Click here for file

Additional file 7 **Definition and overview of constraints for simulations as used in the optimization problems for varied substrate availability.** Constraints listed in this table were applied in simulations for varied substrate availability. For all three simulation settings the corresponding target function and applied constraints are given. The simulation settings include, first, simulations of substrate uptake rates for four different substrates and oxygen demands while satisfying a baseline ATP consumption rate. Second, simulations of substrate uptake rates for four different substrates and oxygen demands as under experimental conditions while satisfying the same baseline ATP consumption rate. Finally, simulations of substrate uptake rates for nine different substrates and oxygen demands while satisfying a predefined metabolic target function. Click here for file

Additional file 8 **Predicted metabolic fluxes of substrate uptake and oxygen demand for ATP expenditure in varied substrate availability,**$\left(C_{i^{+}}>\mathrm{0.6}\right)$**.** We simulated a varied substrate availability for four selected substrates, including glucose, oleate, acetoacetate and lactate while demanding a baseline ATP consumption rate (*v*_ATPase_) of 21.6 mmol·min^−1^·(l cell)^−1^. This table lists uptake rates for oxygen, glucose, oleate, acetoacetate, lactate and the resulting total substrate uptake rate for each simulated substrate composition. Efficiency indices ( $\left(C_{i^{+}}\right)$) were separately calculated for each simulation. Results are shown for calculated efficiency values ( $\left(C_{i^{+}}\right)$) greater than 0.6 and given in descending order. Click here for file

Additional file 9 **Predicted metabolic fluxes of substrate uptake and oxygen demand for ATP expenditure in varied substrate availability,**$\left(C_{i^{+}}<\mathrm{0.6}\right)$**.** See caption of Additional file 8 but results are shown for calculated efficiency values ( $\left(C_{i^{+}}\right)$) equal or less than 0.6. Click here for file

Additional file 10 **Alternate optima.** To identify alternate flux solutions that can equally satisfy the problem, i.e. yield the same optimal solution, we performed additional simulations. The MILP was re-solved after adding a constraint (z*) for a single flux of the original flux distribution which was set to either 1.01-fold ( $\left(z_{1}^{*}\right)$) or 0.99-fold ( $\left(z_{2}^{*}\right)$) of its original calculated flux value (v0). The optimization problem was repeated with substrate combinations which were identified with the highest or lowest efficiency value while satisfying (1) a baseline ATP consumption rate and (2) a target function of the cardiomyocyte. This table includes all calculated flux solutions yielding the same optimal solution as with the original optimization problem. Furthermore, an overview is given of alternate flux solutions for fluxes representing external substrate and oxygen uptake. Statistical significance between flux solutions for the analysis of alternate flux solutions was determined by use of 1-way ANOVA. Click here for file

Additional file 11 **Predicted metabolic fluxes of substrate uptake and oxygen demand for fulfilling the metabolic target function in varied substrate availability,**$\left(C_{i^{+}}>\mathrm{0.8}\right)$**.** We simulated a varied substrate availability for nine selected substrates, including glucose, palmitate, stearate, oleate, alpha-linoleate, eicosapentaenoate, docosahexaenoate, acetoacetate and lactate. During the simulations, we demanded an ATP expenditure (*v*_ATPase_) of 21.6 mmol·min^−1^·(l cell)^−1^ and metabolic target flux, as specified in Additional file 6. This table lists results for substrate combination for which efficiency values ( $\left(C_{i^{+}}\right)$) greater than 0.8 were calculated. Uptake rates for all nine substrates, the resulting total substrate uptake rate and oxygen consumption rate for all simulated substrate compositions which fulfilled the metabolic objective are given. Furthermore solutions for glycogen synthesis and glycogenolysis as determined during simulations are shown. Click here for file

Additional file 12 **Predicted metabolic fluxes of substrate uptake and oxygen demand for fulfilling the metabolic target function in varied substrate availability,**$\left(C_{i^{+}}<\mathrm{0.8}\right)$**.** See caption of Additional file 7 but results are shown for calculated efficiency values ( $\left(C_{i^{+}}\right)$) equal or less than 0.8. Click here for file

Additional file 13 **Gene expression annotation.** The identification of human heart tissue specific reactions requires a tissue specific gene expression profile. We obtained gene expression samples from different gene expression data available from Gene Expression Omnibus, including GDS181 and GSE1145. This table provides gene expression information annotated to metabolic reactions of the cardiomyocyte network. Each reaction identifier refers to a compartment localisation of the respective metabolic reaction. Furthermore, each entry in the table provides information about annotated Ensemble Gene ID, Geo Dataset ID, Geo Sample ID, Probeset ID, Gene ID, gene expression value and detection call. The information of gene expression status can be obtained from the column “Detection call”. Each expression is either categorized as present (P), absent (A) or M (marginal). We further considered genes as expressed for gene expression values with a cut-off greater than 100. Click here for file
